# Scutellarin Alleviates Ovalbumin-Induced Airway Remodeling in Mice and TGF-β-Induced Pro-fibrotic Phenotype in Human Bronchial Epithelial Cells *via* MAPK and Smad2/3 Signaling Pathways

**DOI:** 10.1007/s10753-023-01947-7

**Published:** 2024-01-02

**Authors:** Minfang Li, Dan Jia, Jinshuai Li, Yaqing Li, Yaqiong Wang, Yuting Wang, Wei Xie, Sheng Chen

**Affiliations:** 1 Department of Respiratory Medicine, Shenzhen Traditional Chinese Medicine Hospital, Shenzhen, 518033 China; 2 The Fourth Clinical Medical College of Guangzhou University of Chinese Medicine, Shenzhen, 518033 China; 3 The Second Clinical Medical College of Guangzhou University of Chinese Medicine, Guangzhou, 510120 China; 4 The First Clinical Medical College of Guangzhou University of Chinese Medicine, Guangzhou, 510120 China; 5 Department of Respiratory Medicine, Affiliated Kunshan Hospital of Jiangsu University, Suzhou, 215300 China

**Keywords:** asthma, scutellarin, TGF-β1, ovalbumin, EMT, Smad, MAPK, airway remodeling.

## Abstract

Asthma is a chronic inflammatory disease characterized by airway hyperresponsiveness (AHR), inflammation, and remodeling. Epithelial-mesenchymal transition (EMT) is an essential player in these alterations. Scutellarin is isolated from *Erigeron breviscapus*. Its vascular relaxative, myocardial protective, and anti-inflammatory effects have been well established. This study was designed to detect the biological roles of scutellarin in asthma and its related mechanisms. The asthma-like conditions were induced by ovalbumin challenges. The airway resistance and dynamic compliance were recorded as the results of AHR. Bronchoalveolar lavage fluid (BALF) was collected and processed for differential cell counting. Hematoxylin and eosin staining, periodic acid-Schiff staining, and Masson staining were conducted to examine histopathological changes. The levels of asthma-related cytokines were measured by enzyme-linked immunosorbent assay. For *in vitro* analysis, the 16HBE cells were stimulated with 10 ng/mL transforming growth beta-1 (TGF-β1). Cell migration was estimated by Transwell assays and wound healing assays. E-cadherin, N-cadherin, and α-smooth muscle actin (α-SMA) were analyzed by western blotting, real-time quantitative polymerase chain reaction, immunofluorescence staining, and immunohistochemistry staining. The underlying mechanisms of the mitogen-activated protein kinase (MAPK) and Smad pathways were investigated by western blotting. In an ovalbumin-induced asthmatic mouse model, scutellarin suppressed inflammation and inflammatory cell infiltration into the lungs and attenuated AHR and airway remodeling. Additionally, scutellarin inhibited airway EMT (upregulated E-cadherin level and downregulated N-cadherin and α-SMA) in ovalbumin-challenged asthmatic mice. For *in vitro* analysis, scutellarin prevented the TGF-β1-induced migration and EMT in 16HBE cells. Mechanistically, scutellarin inhibits the phosphorylation of Smad2, Smad3, ERK, JNK, and p38 *in vitro* and *in vivo*. In conclusion, scutellarin can inactivate the Smad/MAPK pathways to suppress the TGF-β1-stimulated epithelial fibrosis and EMT and relieve airway inflammation and remodeling in asthma. This study provides a potential therapeutic strategy for asthma.

## Introduction

Asthma, affecting 262.41 million populations and resulting in 461.07 thousand deaths worldwide in 2019, is a common chronic inflammatory airway disorder [1]. In China, 30 million individuals suffer from asthma, and the incidence appears to be increasing [2, 3]. Clinically, patients with asthma often display recurrent episodes of wheeze, cough, chest tightness, and breath shortness [4]. Asthma can contribute to permanent disability and premature death, and asthma-related costs are high [5, 6]. Currently, inhaled corticosteroids (ICS) are widely used to manage asthma by reducing airway inflammation and inhibiting the development and exacerbations of fixed airflow limitation. However, the side effect profile of ICS, including systemic side effects (reduction in growth velocity, adrenal suppression, osteoporosis, diabetes, respiratory infections, and cataracts) and local side effects (dysphonia, thrush, pharyngitis, and reflex cough), may limit its therapeutic efficacy [7]. Therefore, an improved understanding of asthma pathogenesis is urgently needed to develop highly active anti-asthmatic agents with no or lower toxicity.

Allergens causing infiltration of immune and inflammatory cells in the airways can induce allergic asthma [8]. The involvement of these cells can cause pathological changes such as airway hyperresponsiveness (AHR), airway inflammation, and airway remodeling [9]. Airway inflammation and remodeling are two primary pathological features of asthma [10]. Asthma is subdivided into “type 2-high (T2-high)” asthma and “type 2-low (T2-low)” asthma based on airway inflammation, and the latter form is rarely diagnosed in clinical practice. T2-high asthma, also known as allergic asthma, is featured by hypersecretion of interleukin (IL)-4, IL-5, and IL-13, elevated eosinophil counts, and total IgE and mucus overproduction [11, 12]. Longstanding airway inflammation is responsible for the occurrence of airway remodeling [13]. Increasing evidence has suggested that airway inflammation and remodeling is associated with epithelial-mesenchymal transition (EMT) [14–16]. EMT is a dynamic pathological process in which epithelial cells gradually lose their epithelial features and acquire mesenchymal characteristics [17]. During this process, epithelium cell-cell adhesion is disrupted, and epithelial cells exhibit enhanced migrative ability by downregulating epithelial markers such as E-cadherin and upregulating mesenchymal membrane associated proteins such as N-cadherin and α-smooth muscle actin (α-SMA) [18]. Thus, an urgent need exists to find a potent agent that can inhibit EMT and airway inflammation and remodeling.

Scutellarin, isolated from *Erigeron breviscapus*, possesses anti-oxidant, anti-inflammatory, myocardial protective, and vascular relaxative effects [19–21]. The lung protective effect of scutellarin has been suggested previously. For example, scutellarin can attenuate lipopolysaccharide-induced acute lung injury by suppressing inflammation, apoptosis, oxidative stress, and mitochondrial dysfunction [22]. Additionally, scutellarin can relieve ischemia/reperfusion-stimulated lung injury through its anti-oxidant, anti-inflammatory, and anti-apoptotic effects [23]. Scutellarin also inhibits the production of MUC5AC mucin in airway epithelial cells and displays promising effects on respiratory diseases [24]. Moreover, scutellarin can ameliorate pulmonary and myocardial fibrosis by inhibiting EMT [25, 26]. However, the biological functions of scutellarin in asthma remain unknown.

TGF-β1, a pleiotropic cytokine secreted from infiltrating immune cells and airway epithelial cells, contributes to cellular maturation and differentiation, inflammation, and tissue remodeling [27, 28]. Increasing evidence has revealed that stimulation of TGF-β1 can induce the occurrence of EMT [29–31]. TGF-β signals are transduced mainly by TGF-β receptor-mediated Smad and non-Smad pathways. Upon TGF-β stimulation, regulatory Smads (Smad2 and Smad3) are recruited to TGF-β receptors and activated by phosphorylation. Subsequently, activated regulatory Smads heteroligomerize with Smad4 and translocate to the nucleus [32]. The mitogen-activated protein kinase (MAPK) pathway, including ERK, JNK, and p38 MAPK subfamilies, is a non-Smad pathway and can regulate EMT [33, 34]. Scutellarin has been found to suppress TGF-β1 expression and activation of ERK and p38 to alleviate interstitial fibrosis and cardiac dysfunction [35]. Therefore, this study was designed to investigate the biological functions of scutellarin in asthma and to detect whether the effect of scutellarin was mediated by the Smad and MAPK pathways. We hypothesized that scutellarin might have a potent anti-asthmatic effect. This study might provide novel insights into the understanding of the protective effect of scutellarin against asthma.

## Materials and Methods

### Cell Culture and Treatment

Human bronchial epithelial cells (16HBE; Procell, Wuhan, China) were cultured in Dulbecco’s modified eagle medium (DMEM; Sigma-Aldrich, Shanghai, China) supplemented with 10% fetal bovine serum (FBS), 100 units/mL penicillin, and 100 μg/mL streptomycin at 37 °C in a humidified incubator containing 5% CO_2_. The cells at passage 3 were digested with trypsin to enhance permeability when the cells grew to 80% confluency, followed by being subcultured in 6-well plates (1 × 10^5^ cells) for 24 h. Then, after starvation in serum-free medium for 6–8 h, the 16HBE cells were pretreated with scutellarin (0, 20, 50, or 100 μM; MedChemExpress, Shanghai, China; 98.56% of purity) for 24 h prior to TGF-β1 (10 ng/mL; MedChemExpress) for 24 h. The concentration of TGF-β1 was chosen according to previous studies [36, 37]. The cells were divided into five independent treatment groups: control group, TGF-β1 group (treated with 10 ng/mL TGF-β1 for 24 h), and TGF-β1 + SCU (20, 50, and 100 μM) groups (pretreated with 20, 50, or 100 μM scutellarin for 24 h prior to treatment with 10 ng/mL TGF-β1 for 24 h).

### Cell Viability Assays

The viability of 16HBE cells after incubation with scutellarin at 0, 5, 10, 20, 50, 100, 200, and 400 μM for 24 h or 48 h was assessed by the cell-counting kit-8 (CCK-8) reagent (Beyotime, Shanghai, China). Briefly, the 16HBE cells were incubated in 96-well plates overnight until complete adherence to the walls. After indicated treatment, the medium was removed, and 10 μL CCK-8 solution was added to each well and incubated at 37 °C for 4 h. The optical density was detected using a microplate reader (Molecular Devices, Shanghai, China) at 450 nm.

### Transwell Assay

Transwell chambers (8 μm pore) obtained from Corning Inc. (Corning, NY, USA) were used to estimate the migrative ability of 16HBE cells. Briefly, after indicated treatment, the 16HBE cells (2 × 10^4^) were seeded into the upper chamber in 200 μL DMEM without FBS, followed by addition of 800 μL DMEM containing 10% FBS into the lower chamber. After incubation for 24 h or 48 h, the 16HBE cells that transferred to the lower chamber were fixed with 4% paraformaldehyde, stained with 0.5% crystal violet (Beyotime) for 5 min, and then counted in five randomly selected fields of view using a light microscope (Olympus, Tokyo, Japan).

### Wound Healing Assays

The 16HBE cells were seeded into 6-well plates (2 × 10^4^ cells/well). When the cells grew to a confluent monolayer, a straight scratch wound in the center of cell monolayer was created with a 200-μL pipette tip. After the cells were washed with phosphate-buffered saline (PBS), the cells were incubated with serum-free DMEM. Images were taken at 0, 24, and 48 h using a light microscope. The migrative ability of 16HBE cells was examined by assessing the distance traveled toward wound center.

### Real-Time Quantitative Polymerase Chain Reaction (RT-qPCR)

Total RNA was isolated from 16HBE cells and lung tissues using TRIzol reagent (Beyotime) and quantified by NanoDrop (Thermo Fisher Scientific, Shanghai, China). The reverse transcription of RNA into cDNA was performed using the BeyoRT^™^ first strand cDNA synthesis kit (Beyotime). Then, the cDNA was amplified by RT-qPCR in a reaction containing SYBR Green PCR Master Mix (Solarbio, Beijing, China) and 0.2 μM primers and analyzed using the ABI 7500 Fast Real-Time PCR System (Thermo Fisher Scientific). The PCR conditions were as follows: initial denaturation at 95 °C for 5 min, followed by 33 cycles of denaturation at 95 °C for 40 s, primer annealing at 52 °C for 30 s, and extension at 72 °C for 25 s. The final extension was conducted at 72 °C for 10 min. Data were normalized to GAPDH and expressed as fold change over control. The 2^−△△Ct^ method was used to determine the relative mRNA folding changes [38]. The primers used in this study are listed in Table 1.

**Table 1:** Sequences of Primers Used for Reverse Transcription-Quantitative PCR

Gene (mice)	Sequence (5′→3′)
E-cadherin forward	AAAAGAAGGCTGTCCTTGGC
E-cadherin reverse	GAGGTCTACACCTTCCCGGT
N-cadherin forward	CCTCCAACGGGCATCTTCAT
N-cadherin reverse	TGTCCACTGCATGTGCTCTC
α-SMA forward	CCCAACTGGGACCACATGG
α-SMA reverse	TACATGCGGGGGACATTGAAG
GAPDH forward	ATGCAACGGATTTGGTCGTAT
GAPDH reverse	TCTCCTCCTGGAAGATGGTG

### Western Blotting

Total protein was isolated from mouse lung tissues and 16HBE cells using RIPA lysis buffer (Sigma-Aldrich) containing 1% protease inhibitor (ApexBio Technology, Shanghai, China), and the protein content was quantified with an Enhanced BCA Protein Assay Kit (Yeasen, Shanghai, China). Then, protein samples (30 μg/group) were separated by sodium dodecyl sulfate polyacrylamide gel electrophoresis (SDS-PAGE) and transferred onto polyvinylidene fluoride (PVDF) membranes. After blocking using 5% skimmed milk for 2 h, the membranes were incubated overnight with primary antibodies against phosphorylated Smad2 (ab280888, 1:1000; Abcam, Shanghai, China), phosphorylated Smad3 (ab52903, 1:2000; Abcam), Smad2/3 (ab202445, 1:1000; Abcam), β-actin (ab6276, 1:5000; Abcam), phosphorylated ERK (sc-135760, 1:1000; Santa Cruz Biotechnology, Shanghai, China), Smad4 (ab40759, 1:5000; Abcam), ERK (sc-398015, 1:500; Santa Cruz Biotechnology), p38 (ab170099, 1:2500; Abcam), phosphorylated JNK (sc-6254, 1:1000; Santa Cruz Biotechnology), JNK (sc-7345, 1:250; Santa Cruz Biotechnology), phosphorylated p38 (ab195049, 1:1000; Abcam), E-cadherin (sc-8426, 1:1000; Santa Cruz Biotechnology), α-SMA (ab5694, 1:500; Abcam), and N-cadherin (ab76011, 1:5000; Abcam) at 4 °C. Subsequently, the membranes were washed three times using TBST and incubated with the corresponding secondary antibodies for 2 h at room temperature. After washing three times with TBST, the protein bands were developed using an enhanced chemiluminescence reagent (Yeasen), and the intensity was quantified by ImageJ software.

### Immunofluorescence Staining

The 16HBE cells were seeded onto 6-well plates (1 × 10^5^ cells/well) and incubated with indicated treatment. The cells were then fixed with 4% paraformaldehyde for 20 min and permeabilized with 0.1% TritonX-100 (Sigma-Aldrich) for 20 min at room temperature. After being blocked with 3% bovine serum albumin (BSA; Sigma-Aldrich) for 1 h, the cells were incubated with primary antibodies against E-cadherin, N-cadherin, and α-SMA at 4 °C overnight. After washing three times using PBS, the cells were incubated with goat anti-mouse conjugated with Alexa 594 secondary antibodies (1:1000; Abcam) at room temperature for 2 h. DAPI (Solarbio) was used to stain the nuclei. Images were taken using a fluorescent microscope (Olympus).

### Co-immunoprecipitation (Co-IP) Assay

Co-IP assay was performed using the Co-IP kit (Thermo Fisher Scientific) according to the manufacturer’s protocol. Total cellular protein extractions from 16HBE cells were immunoprecipitated using anti-SMAD2/3 antibody. The anti-SMAD4 antibody was used as the detecting antibody. The samples were analyzed using the western blotting procedures.

### Animals and Ethics Statement

Female and male BALB/c mice (6 weeks of age, weighing 20 ± 2 g) were obtained from Charles River Laboratories (Beijing, China) and housed under standard conditions (22 ± 2 °C, 50–60% of humidity, 12-h light/dark cycle). All animals were given free access to food and water. Animal experiments complied with the guidelines for the care and use of experimental animals of the International Association for Assessment and Accreditation of Laboratory Animal Care. Animal ethics approval was obtained from the Ethics Committee of Shenzhen Traditional Chinese Medicine Hospital.

### Experimental Groups

The mice were randomly allocated to six groups (each group had 10 mice, 5 females and 5 males): (1) control group, saline challenge and saline treatment; (2) OVA group, ovalbumin challenge and saline treatment; (3) OVA + SCU-L group, ovalbumin challenge and 30 mg/kg scutellarin treatment; (4) OVA + SCU-M group, ovalbumin challenge and 60 mg/kg scutellarin treatment; (5) OVA + SCU-H group, ovalbumin challenge and 90 mg/kg scutellarin treatment; (6) OVA + DEX group, ovalbumin challenge and 1 mg/kg dexamethasone treatment. The doses of scutellarin were given as previously documented [25].

### Ovalbumin-Induced Asthma Model

The mice were acclimated under standard conditions for 1 week before experiments. The experimental asthma model was established as previously described [39]. Briefly, the mice were sensitized with intraperitoneal injection of 10 μg ovalbumin (grade V; Sigma-Aldrich) in 0.2 mL aluminum hydroxide gel (Sigma-Aldrich) on days 0 and 14. After 2 weeks, the mice were given aerosol challenges with 1% ovalbumin for 20 min using an ultrasonic nebulizer (Omron Co., Tokyo, Japan) twice a day for successive 8 weeks. The control mice were injected with 0.2 mL aluminum hydroxide gel and challenged with saline in a similar manner. From day 28, the ovalbumin-sensitized mice were orally administrated with 0.2 mL saline containing dexamethasone (positive control; 1 mg/kg; MedChemExpress) or scutellarin (30, 60, or 90 mg/kg; MedChemExpress) 1 h before each ovalbumin challenge for 8 weeks, whereas the control mice were administrated with normal saline. The mice were sacrificed by cervical dislocation 24 h after the final challenge. The schematic diagram of the drug treatment is shown in Fig. 4a.

### Measurement of AHR in Mice

The mice were placed in the EMKA animal lung function test system (GYD Labtech, Beijing, China) 24 h after the final challenge. After the mice stably breathed, they were atomized inhalation with increasing doses of methacholine solution (0.0625, 0.125, 0.25, 0.5, 1, and 2 μg/mL, 50 μL each time) following manufacturer’s instructions. Airway resistance (Raw) and dynamic compliance (Cdyn) of mice were recorded as the AHR results.

### Analysis of Bronchoalveolar Lavage Fluid (BALF)

The mice were sacrificed 24 h after the final challenge, and the left bronchus was ligated, with the right bronchus being lavaged three times with 750 μL precooled PBS. The fluid recovery rate was 80%, and the input volume was defined as BALF. Thereafter, the BALF was centrifuged for 10 min at 500 × *g* at 4 °C, and a hemocytometer (Thermo Fisher Scientific) was used to count the number of total leukocytes in the BALF. For differential cell counting, the cell smears were stained with Wright-Giemsa (Beyotime) and were counted by two independent blinded investigators.

### Histopathological Assessment

The left lung tissues were removed after collection of BALF and fixed with 4% paraformaldehyde for 24 h, dehydrated, paraffin-embedded, and cut into 5-μm sections. The sections were then stained with hematoxylin and eosin (HE; Sigma-Aldrich), periodic acid-Schiff (PAS) solution (Sigma-Aldrich), and Masson trichrome (Sigma-Aldrich) to evaluate airway inflammation, mucus production, and collagen deposition in the lung tissues. The airway inflammation and remodeling changes were observed using a light microscope. The inflammation score was quantified by three independent individuals blinded to the experimental methods and ranged from 0 to 3: 0, no inflammatory cells; 1, focal infiltration of inflammatory cells around the airway; 2, multiple infiltration of inflammatory cells around the airway; 3, massive infiltration of inflammatory cells around the airway [40].

### Enzyme-Linked Immunosorbent Assay (ELISA)

The lung tissues collected from mice were stripped and perfused with PBS. Then, the tissues were homogenized and centrifuged at 3000 r/min for 20 min, followed by collection of the supernatants. The blood of retro-orbital plexus of mice was collected and centrifuged at 3000 × *g* for 10 min, followed by collection of the serum. The levels of IL-4, IL-5, IL-13, eotaxin, TGF-β, and MUC5AC in the lung and the level of IgE in serum of mice were measured using commercially available ELISA kits (Enzyme-linked Biotechnology, Shanghai, China) according to the manufacturer’s instructions.

### Immunohistochemistry Staining

The left lungs were collected and fixed in 4% paraformaldehyde, embedded in paraffin, and cut into 5-μm sections. After deparaffinization and rehydration, the lung sections were incubated with 3% hydrogen peroxide in methanol for 30 min to block the endogenous peroxidase activity. After being blocked with 10% BSA in PBS for 1 h, the sections were incubated with primary antibodies against E-cadherin (sc-8426, 1:250; Santa Cruz Biotechnology), N-cadherin (ab76011, 1:500; Abcam), and α-SMA (ab5694, 1:100; Abcam) overnight at 4 °C, followed by incubation with HRP-conjugated goat anti-rabbit IgG secondary antibodies. Peroxidase conjugates were visualized using DAB solution (Yeasen). The sections were counterstained with hematoxylin and mounted on a coverslip. The images were taken using a microscope (Olympus) and quantified by Image-Pro Plus software.

### Statistical Analysis

All experiments were performed at least three independent repeats. Statistics were analyzed using GraphPad Prism 8 (GraphPad Software, San Diego, CA, USA). Data were described as the mean ± standard deviation. The comparisons among multiple groups were analyzed by one-way analysis of variance followed by Tukey’s *post hoc* analysis. *p* < 0.05 was considered statistically significant.

## Results

### Scutellarin Suppresses the TGF-β1-Stimulated Migration of 16HBE Cells

**Fig. 1:**
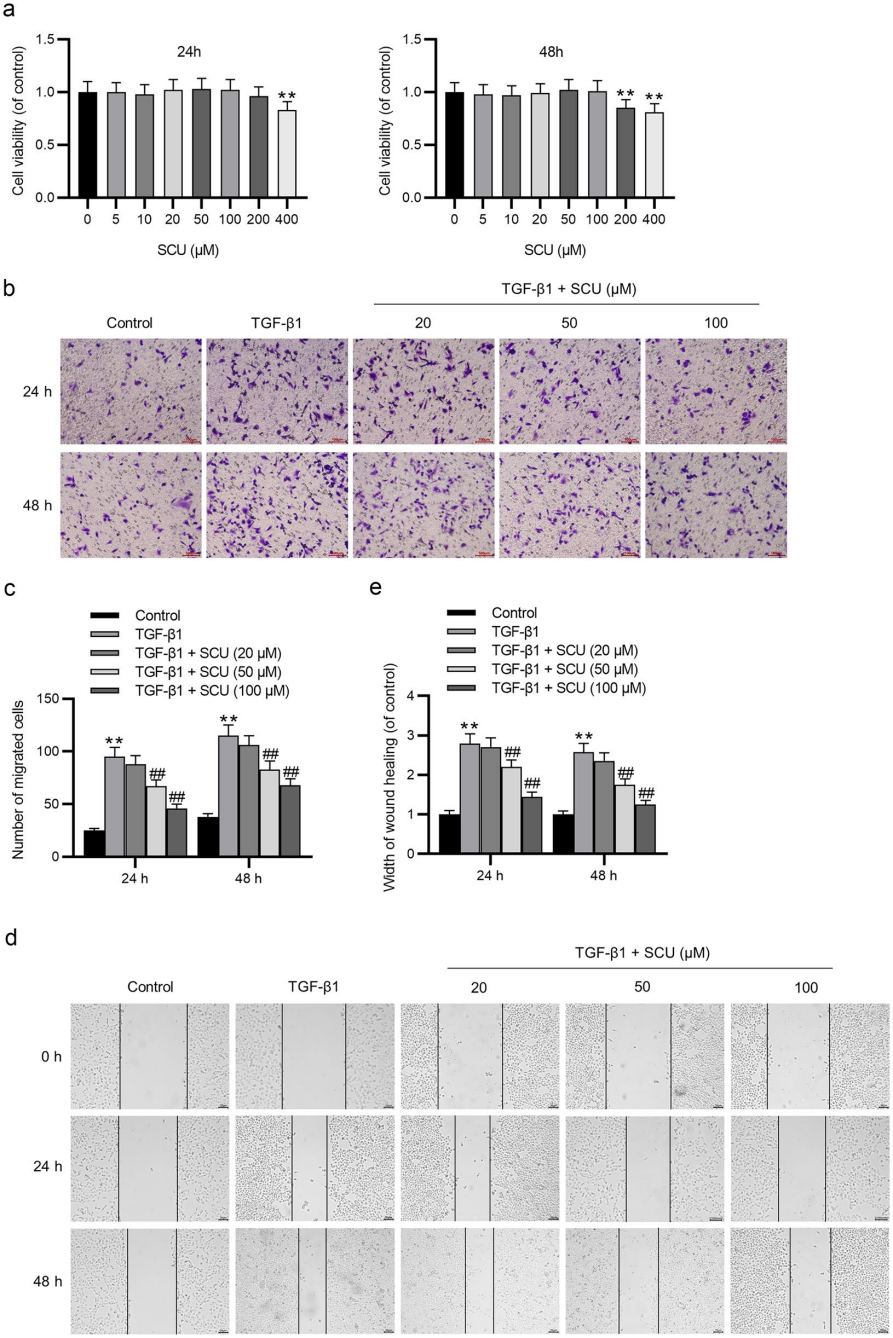
Scutellarin inhibits the TGF-β1-induced migration of 16HBE cells. **a** The 16HBE cells were treated with 0–400 μM scutellarin for 24 h and 48 h, and the effect of scutellarin on the viability of 16HBE cells was detected by CCK-8 assays. **p < 0.01 vs. the SCU (0 μM group). **b**–**e** Cell migration was detected by Transwell assays and wound healing assays at 24 h and 48 h. Data are analyzed by one-way analysis of variance followed by Tukey’s *post hoc* analysis and expressed as the mean ± standard deviation of three independent experiments. **p < 0.01 vs. the control group, ^##^*p* < 0.01 vs. the TGF-β1 group.

The cytotoxic effect of scutellarin at different concentrations was examined by CCK-8 assays. The results showed that low doses of scutellarin (0, 5, 10, 20, 50, and 100 μM) had no significant cytotoxicity to 16HBE cells, whereas scutellarin at 400 μM significantly suppressed cell viability after 24 h of incubation and scutellarin at 200 and 400 μM resulted in a significant reduction in cell viability after 48 h of incubation (Fig. 1a). Subsequently, the effect of scutellarin at 20, 50, and 100 μM on the migrative capacity of TGF-β1-exposed 16HBE cells was examined using Transwell assays and wound healing assays. The results revealed that TGF-β1 significantly enhanced the migrative capability of 16HBE cells, whereas the enhancing effect was limited by scutellarin dose dependently after 24 h and 48 h of incubation (Fig. 1b, c). The increased wound closure ability induced by TGF-β1 was suppressed by scutellarin in a dose-dependent manner (Fig. 1d, e). Collectively, scutellarin treatment attenuated the enhancing effect of TGF-β1 on the migration of 16HBE cells.

### Scutellarin Ameliorates the TGF-β1-Stimulated EMT in 16HBE Cells

**Fig. 2:**
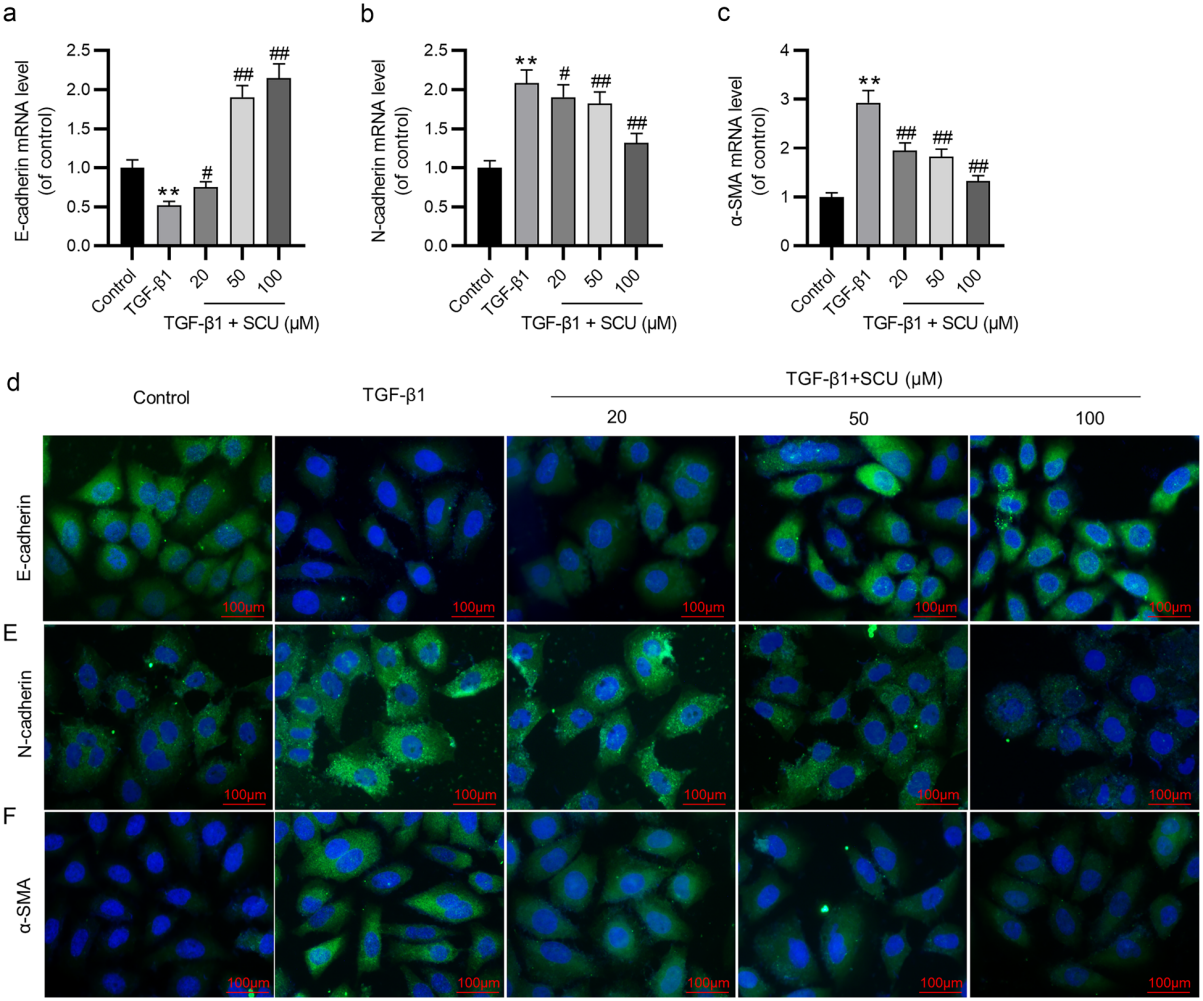
Scutellarin ameliorates the TGF-β1-induced EMT in 16HBE cells. **a**–**c** The mRNA levels of E-cadherin, N-cadherin, and α-SMA were measured by RT-qPCR. **d**–**f** The expression of E-cadherin, N-cadherin, and α-SMA was estimated by immunofluorescence staining. The 16HBE cells were stained with E-cadherin, N-cadherin, and α-SMA (green) and nuclei were stained with DAPI (blue). Fluorescent images were taken using a fluorescent microscope. Data are analyzed by one-way analysis of variance followed by Tukey’s *post hoc* analysis and expressed as the mean ± standard deviation of three independent experiments. **p < 0.01 vs. the control group; ^#^*p* < 0.05, ^##^*p* < 0.01 vs. the TGF-β1 group.

EMT is an essential step in airway remodeling. Downregulation of E-cadherin and upregulation of α-SMA and N-cadherin are hallmarks of EMT [41]. Thus, we measured the expression of E-cadherin, α-SMA, and N-cadherin by RT-qPCR and immunofluorescence staining. The results of RT-qPCR demonstrated that the mRNA level of E-cadherin was significantly decreased, whereas the mRNA levels of N-cadherin and α-SMA were significantly increased after TGF-β1 treatment. However, treatment with scutellarin had the opposite effect (Fig. 2a–c). Similarly, as shown by immunofluorescence staining, TGF-β1 significantly reduced E-cadherin protein level while remarkably elevating N-cadherin and α-SMA expression. However, the above effect was effectively abolished by scutellarin in a dose-dependent manner (Fig. 2d–f). These results suggest that scutellarin suppresses EMT in TGF-β1-exposed 16HBE cells.

### Scutellarin Inactivates the Smad and MAPK Pathways in 16HBE Cells

**Fig. 3:**
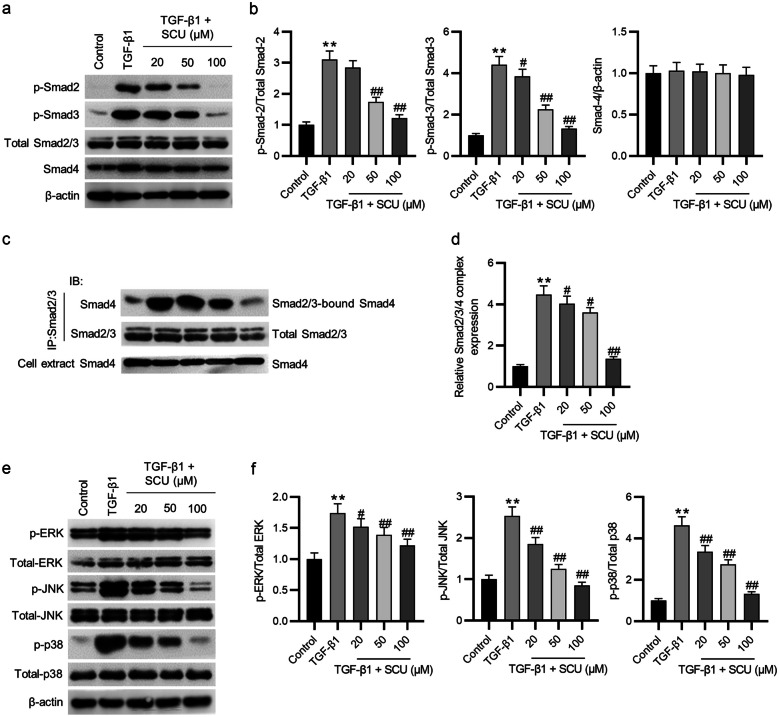
Scutellarin inactivates the Smad and MAPK pathways in TGF-β1-treated 16HBE cells. **a**, **b** The protein levels of phosphorylated Smad2/Smad3 and total Smad4 were evaluated by western blotting. **c**, **d** The effect of scutellarin on the formation of Smad2/3/4 complex in TGF-β1-treated 16HBE cells was examined by co-immunoprecipitation. **e**, **f** The protein levels of phosphorylated ERK, phosphorylated JNK, and phosphorylated p38 were measured by western blotting. Data are analyzed by one-way analysis of variance followed by Tukey’s *post hoc* analysis and expressed as the mean ± standard deviation of three independent experiments. **p < 0.01 vs. the control group; ^#^*p* < 0.05, ^##^*p* < 0.01 vs. the TGF-β1 group.

To detect the mechanisms by which scutellarin suppressed the TGF-β1-stimulated EMT, Smad and MAPK pathways in 16HBE cells were assessed by western blotting. Scutellarin effectively counteracted the enhancing effect of TGF-β1 on the phosphorylation levels of Smad2 and Smad3 dose dependently, while the protein level of Smad4 had no obvious change (Fig. 3a, b). Then, we used Smad2/3 antibody to absorb all Smad2/3 proteins in cell supernatant by immunoprecipitation and evaluated the protein level of Smad4 in the immunoprecipitated samples by Smad4 antibody. The results showed that TGF-β1 notably enhanced the formation of Smad2/3/4 complex, which was inhibited by scutellarin dose dependently (Fig. 3c, d). Finally, as shown by western blotting, the TGF-β1-induced increase in the phosphorylation levels of ERK, JNK, and p38 was inhibited by scutellarin (Fig. 3e, f). Taken together, scutellarin inactivates the Smad and MAPK pathways in TGF-β1-treated 16HBE cells.

### Scutellarin Relieves the Ovalbumin-Induced AHR in Mice

**Fig. 4:**
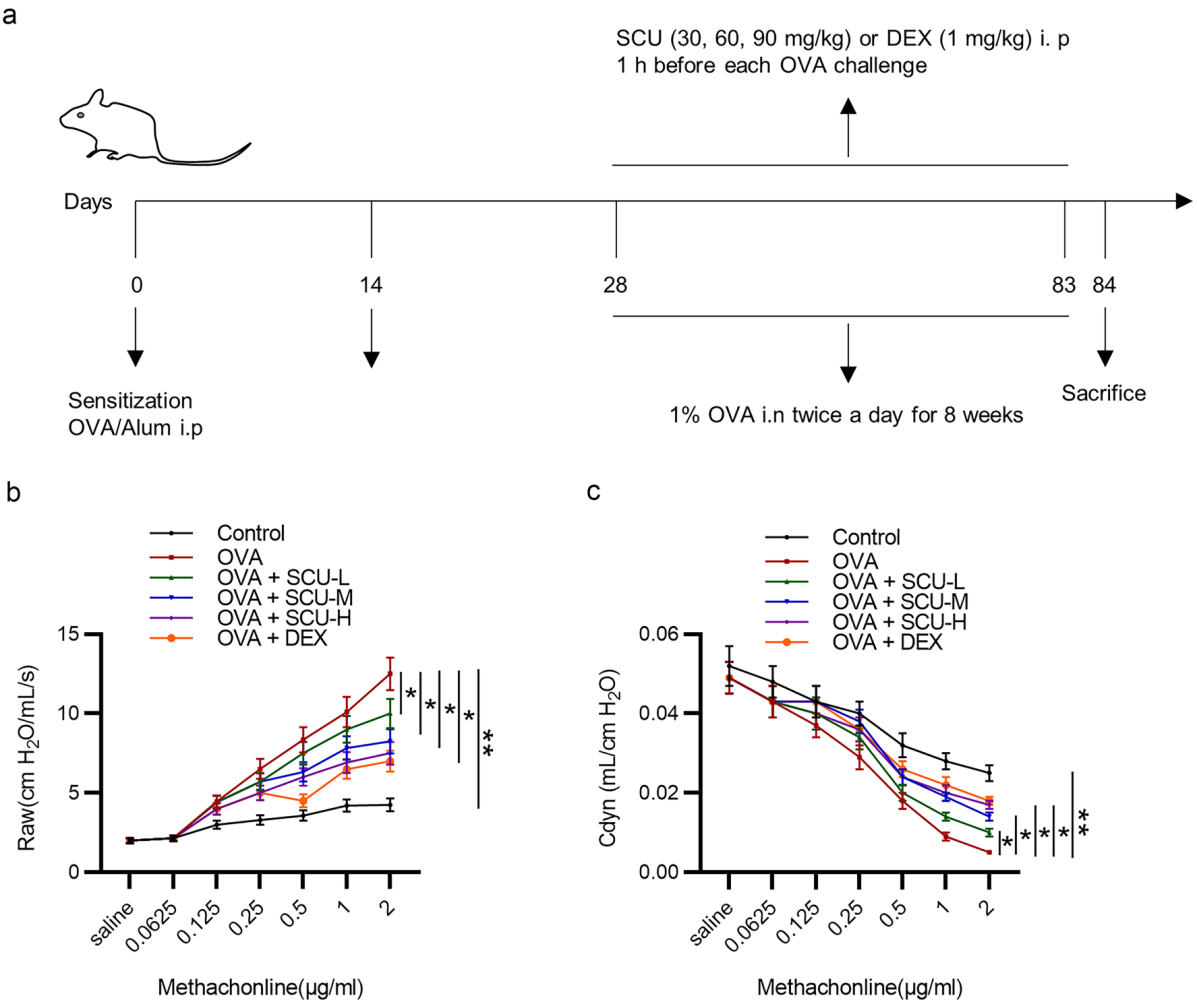
Scutellarin relieves the ovalbumin-induced airway hyperresponsiveness in mice. **a** The experimental design for asthma model. **b** The effect of scutellarin on Raw (*n* = 6). **c** The effect of scutellarin on Cdyn in the ovalbumin-challenged asthmatic mice (*n* = 3 mice each group). Data are analyzed by one-way analysis of variance followed by Tukey’s *post hoc* analysis and expressed as the mean ± standard deviation. *p < 0.05, **p < 0.01.

Ovalbumin-challenged asthmatic mouse model is commonly used to investigate human asthma [42]. Here, we established an ovalbumin-challenged asthmatic mouse model to detect the therapeutic efficacy of scutellarin in the management of asthma. AHR refers to a characteristic feature of asthma. The OVA group exhibited a remarkably higher Raw than the control group, whereas administration of scutellarin counteracted the ovalbumin-induced promotion in Raw dose dependently, and dexamethasone had the similar effect with scutellarin (Fig. 4b). Additionally, the Cdyn was markedly downregulated in the OVA group, while administration of scutellarin or dexamethasone attenuated the suppressive effect of ovalbumin on Cdyn (Fig. 4c). Collectively, the AHR of ovalbumin-induced allergic asthmatic mice is relieved by scutellarin.

### Scutellarin Alleviates the Ovalbumin-Stimulated Airway Inflammation and Remodeling in Mice

**Fig. 5:**
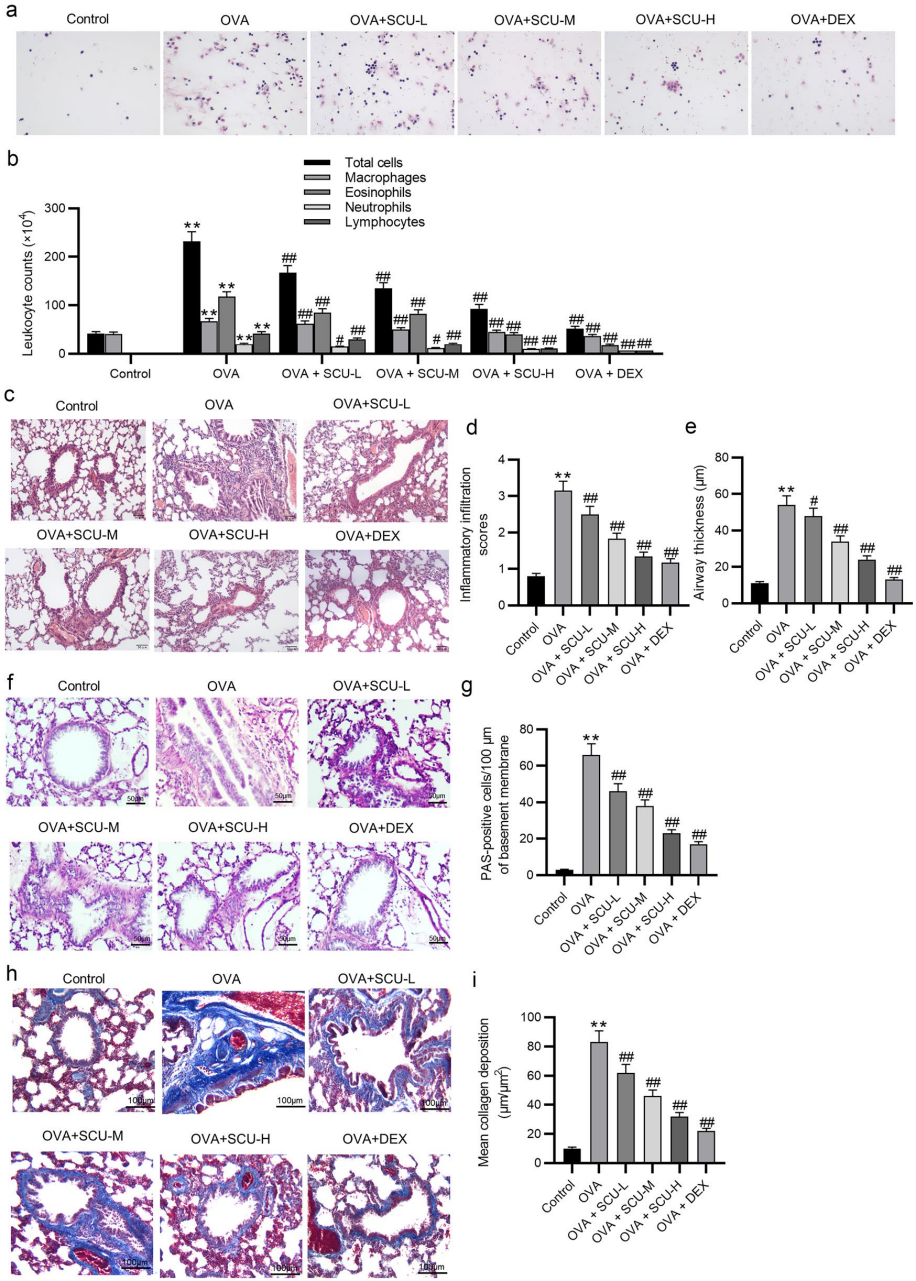
Scutellarin alleviates the ovalbumin-induced airway inflammation and remodeling in mice. **a** In the results of Wright-Giemsa staining, the blue arrows point to the macrophages, green arrows to the eosinophils, and yellow arrows to the lymphocytes (*n* = 6). **b** The leukocyte counts of BALF. **c** Lung histopathologic changes were examined by HE staining (*n* = 6). **d** Total inflammation scores. **e** Quantified results of airway wall thickness. **f**, **g** PAS staining was used to measure mucus production (*n* = 6). **h**, **i** Collagen deposition was evaluated by Masson staining (*n* = 6). Data are analyzed by one-way analysis of variance followed by Tukey’s *post hoc* analysis and expressed as the mean ± standard deviation. **p < 0.01 vs. the control group; ^#^*p* < 0.05, ^##^*p* < 0.01 vs. the OVA group.

The development of airway inflammation and remodeling was evaluated by leukocyte counts of BALF and histopathological analysis of lung lesions. The results of Wright-Giemsa staining revealed that the number of total inflammatory cells, macrophages, eosinophils, neutrophils, and lymphocytes was significantly higher in ovalbumin-induced asthmatic mice, which was decreased by scutellarin or dexamethasone (Fig. 5a, b). Then, as shown by HE staining, peribronchial infiltration of inflammatory cells was caused by ovalbumin, whereas treatment with scutellarin or dexamethasone abolished the ovalbumin-induced inflammatory changes around the bronchus (Fig. 5c, d). Additionally, the ovalbumin-challenged mice displayed significantly increased thickness of peribronchial smooth muscle layer compared with controls, which was reduced by scutellarin or dexamethasone (Fig. 5e). PAS staining was performed to evaluate the epithelial changes, including goblet cell hyperplasia and mucus production. The results revealed that ovalbumin caused goblet cell hyperplasia in the mucosal epithelium and mucus overproduction in the airway lumen, whereas scutellarin or dexamethasone treatment remarkedly reduced the PAS-stained positive area (Fig. 5f, g). Moreover, as Masson staining revealed, the increase of collagen deposition caused by ovalbumin was limited by scutellarin or dexamethasone (Fig. 5h, i). These results suggest that scutellarin may reduce the ovalbumin-stimulated airway inflammation and remodeling in mice.

### Scutellarin Reduces the Expression of Asthma-Related Cytokines

**Fig. 6:**
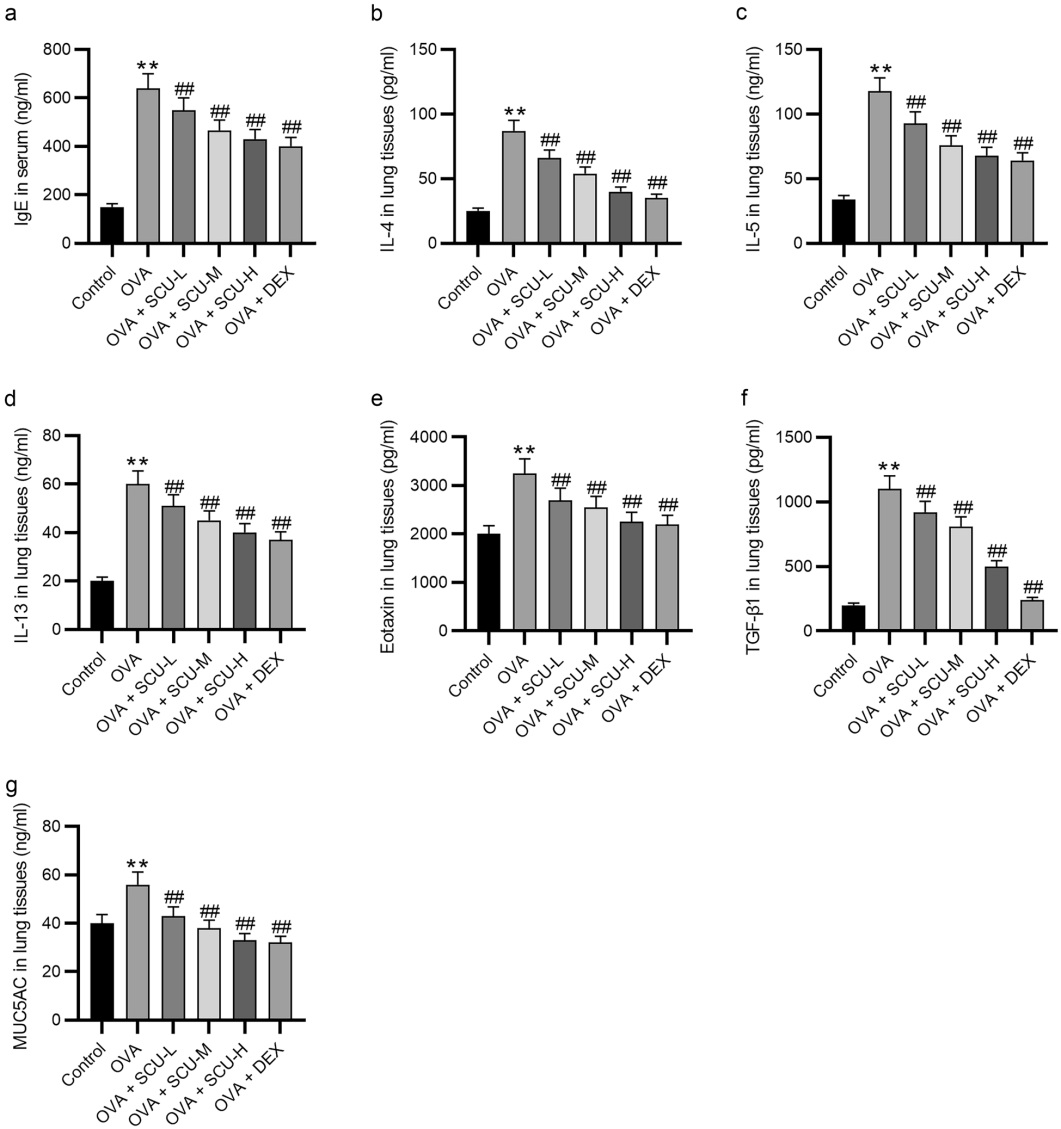
Scutellarin reduces the levels of asthma-related cytokines. **a** The IgE level in the serum (*n* = 6). **b**–**g** The levels of IL-4, IL-5, IL-13, eotaxin, TGF-β1, and MUC5AC in the lung tissues of ovalbumin-challenged asthmatic mice were measured by ELISA (*n* = 6). Data are analyzed by one-way analysis of variance followed by Tukey’s *post hoc* analysis and expressed as the mean ± standard deviation. **p < 0.01 vs. the control group, ^##^*p* < 0.01 vs. the OVA group.

Then, we assessed the levels of IgE, Th2 cytokines (IL-4, IL-5, and IL-13), eotaxin, TGF-β1, and MUC5AC using ELISA kits to detect the effect of scutellarin on the release of asthma-related cytokines in ovalbumin-challenged mice. The levels of these cytokines were remarkably increased following ovalbumin challenges. However, administration of scutellarin or dexamethasone significantly suppressed the ovalbumin-induced upregulation (Fig. 6a–g).

### Scutellarin Represses EMT in Lung Tissues of Ovalbumin-Challenged Asthmatic Mice

**Fig. 7:**
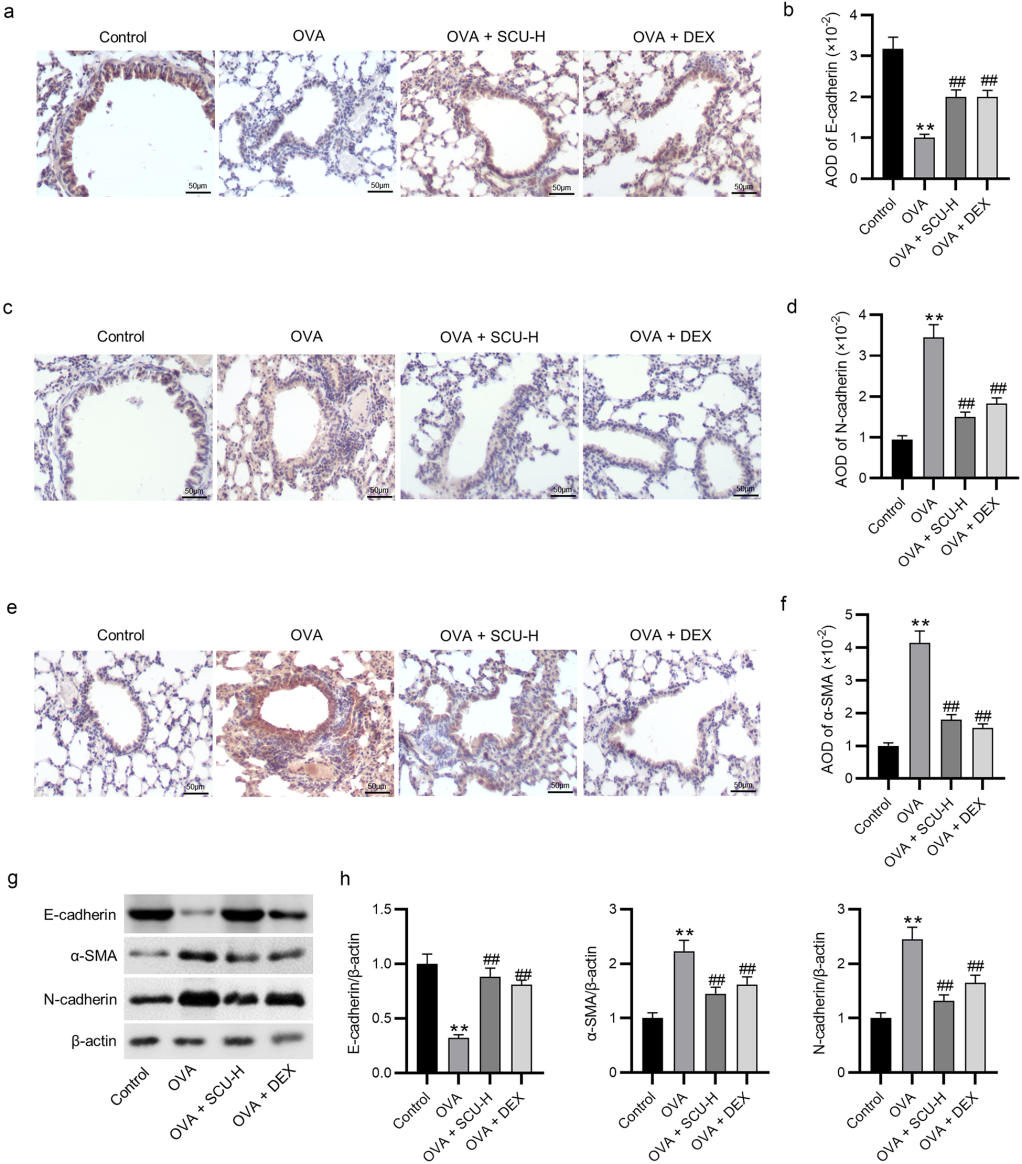
Scutellarin represses EMT in lung tissues of ovalbumin-challenged asthmatic mice. **a**–**f** The expression of E-cadherin, N-cadherin, and α-SMA was measured by immunohistochemistry staining (*n* = 6). **g**, **h** Western blotting was performed to evaluate the protein levels of E-cadherin, N-cadherin, and α-SMA (*n* = 6). Data are analyzed by one-way analysis of variance followed by Tukey’s *post hoc* analysis and expressed as the mean ± standard deviation. **p < 0.01 vs. the control group, ^##^*p* < 0.01 vs. the OVA group.

As shown by immunohistochemistry staining, E-cadherin level was decreased but the levels of N-cadherin and α-SMA were increased in the lungs of the ovalbumin-challenged asthmatic mice, whereas treatment with scutellarin or dexamethasone had the opposite effect (Fig. 7a–f). Additionally, scutellarin or dexamethasone rescued the decreased E-cadherin protein level but reversed the promotion in the protein levels of α-SMA and N-cadherin in the lungs of mice with ovalbumin-induced asthma (Fig. 7g, h). Taken together, scutellarin suppresses the EMT event in the ovalbumin-challenged asthmatic mice.

### Scutellarin Inactivates the Smad and MAPK Pathways in the Lungs of OVA-Challenged Asthmatic Mice

**Fig. 8:**
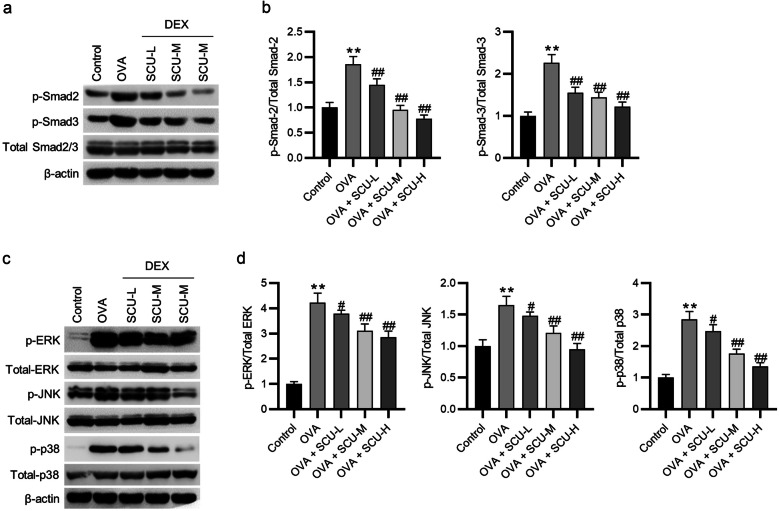
Scutellarin inactivates the Smad and MAPK pathways in lung tissues of ovalbumin-challenged asthmatic mice. **a**, **b** The phosphorylation of Smad2 and Smad3 was estimated by western blotting. **c**, **d** The phosphorylation of ERK, JNK, and p38 was measured by western blotting (*n* = 6). Data are analyzed by one-way analysis of variance followed by Tukey’s *post hoc* analysis and expressed as the mean ± standard deviation. **p < 0.01 vs. the control group; ^#^*p* < 0.05, ^##^*p* < 0.01 vs. the OVA group.

**Fig. 9:**
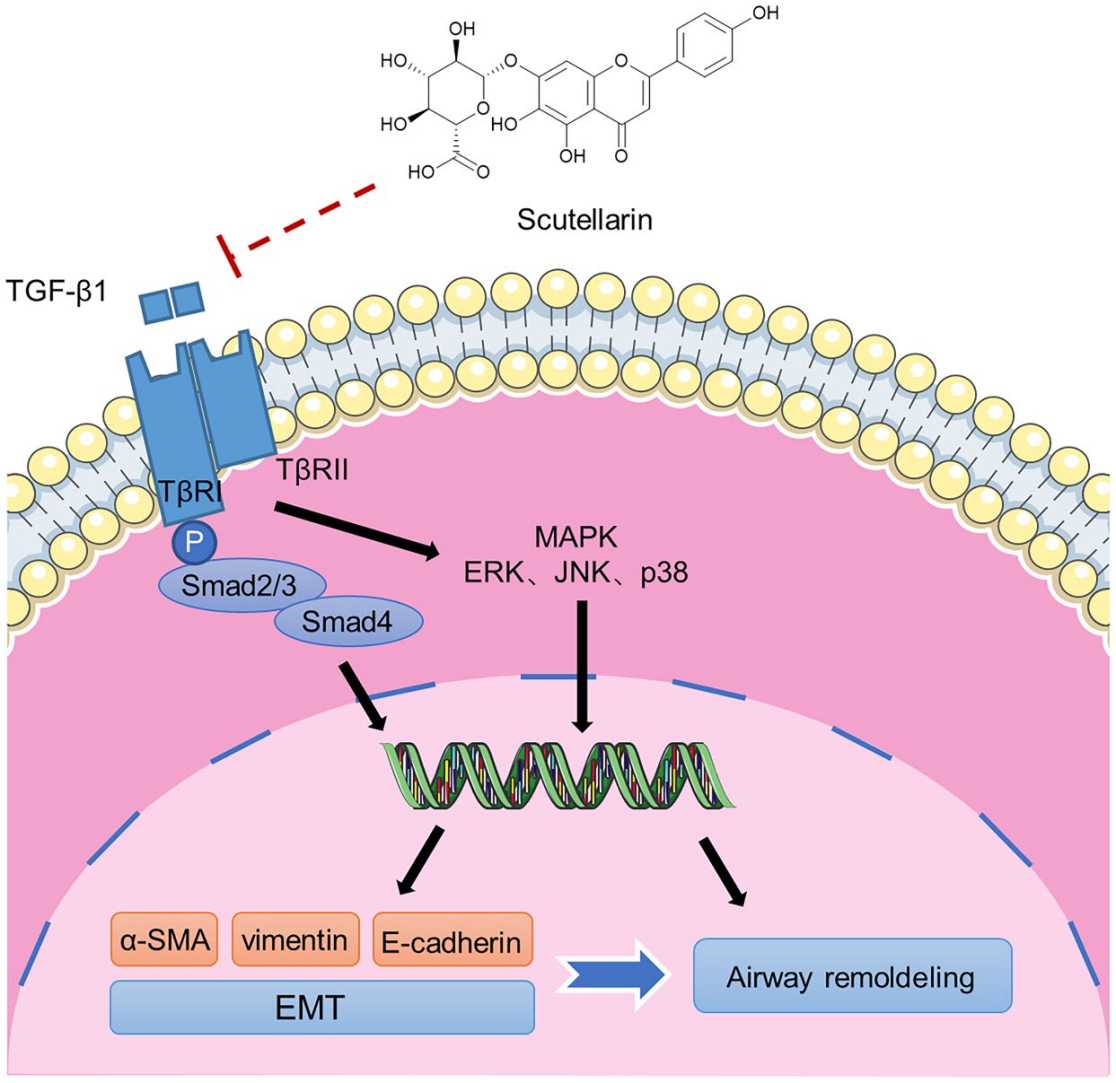
Scientific diagram depicting the mechanisms by which scutellarin alleviates the TGF-β1-induced EMT process.

The Smad and MAPK pathways in lung tissues were assessed by western blotting. The results revealed that the phosphorylation levels of Smad2, Smad3, ERK, JNK, and p38 were markedly augmented in the lung tissues of ovalbumin-challenged asthmatic mice, whereas administration of scutellarin notably prevented their phosphorylation (Fig. 8a–d). Figure 9 presents the schematic diagram depicting the mechanisms by which scutellarin inhibits EMT in TGF-β1-treated 16HBE cells. Scutellarin can inhibit the TGF-β1-induced EMT by inhibiting the Smad and MAPK pathways.

## Discussion

Asthma is a chronic inflammatory disease characterized by AHR, airway inflammation, and airway remodeling, with an increasing morbidity and mortality. Current strategies for the management of asthma cannot cure asthma and have side effects. Hence, there is an urgent need to explore more effective therapeutic options for treating asthma, Scutellarin, a flavonoid glucuronide isolated from *Erigeron breviscapus*, is well known for its anti-oxidant, anti-inflammatory, anti-thrombotic, and anti-apoptotic actions [43–45]. In the current study, we used TGF-β1-treated 16HBE cells and ovalbumin-challenged asthmatic mice to investigate the biological functions of scutellarin in the pathogenesis of airway inflammation and remodeling and related mechanisms. Scutellarin could relieve airway inflammation and remodeling by inhibiting the EMT event as indicated by the following findings: (a) scutellarin inhibited the TGF-β1-induced migration and EMT of 16HBE cells; (b) scutellarin alleviated the ovalbumin-induced AHR, airway remodeling, and airway inflammation in mice; (c) scutellarin inhibited the EMT process in the lung tissues of ovalbumin-challenged asthmatic mice; and (d) scutellarin inactivated the Smad2/Smad3 and MAPK pathways *in vivo* and *in vitro*.

As reported, airway remodeling refers to airway structural alternations characterized by subepithelial fibrosis, airway wall thickening, subepithelial collagen deposition, and excessive mucus secretion, causing AHR and airway obstruction [46–48]. Mucus hypersecretion of the MUC5AC by goblet cells is also a pathophysiologic feature of airway remodeling in asthmatics [49]. Scutellarin is found to inhibit MUC5AC mucin production induced by human neutrophil elastase on airway epithelial cells *via* ERK-dependent and protein kinase C-dependent pathways [24, 50]. Additionally, scutellarin can decrease the collagen fiber deposition in the thickened interalveolar septa and prevent the pulmonary edema to ameliorate lung injury in mice [23]. In the current study, we found that ovalbumin-challenged asthmatic mice displayed airway wall thickening, mucus hypersecretion, and collagen deposition, whereas administration of scutellarin attenuated these pathological changes, suggesting that scutellarin can effectively inhibit airway remodeling in experimental asthmatic models.

Chronic inflammation in chronic asthma can cause airway remodeling. The infiltration of inflammatory cells induces mucus overproduction and structural damage [51]. In asthmatic patients, airway inflammation usually involves Th2 cells that release Th2 cytokines (IL-4, IL-5, and IL-13) to modulate inflammatory response. Th2 cytokines are essential for IgE synthesis, chemokine production, smooth muscle hyperplasia, mucus production, and airway eosinophilia [52–54]. Eosinophils are critical players in tissue remodeling. They promote myofibroblast maturation, fibroblast proliferation, and collagen synthesis and constitute the main source of TGF-β [55, 56]. Eotaxin, produced by epithelial cells, endothelial cells, and lung fibroblasts, is an efficient eosinophil chemoattractant [57]. The current study demonstrated that ovalbumin challenges increased the number of inflammatory cells, particularly eosinophils, in the BALF. Additionally, the levels of IgE, Th2 cytokines, eotaxin, and TGF-β1 were also remarkably upregulated after ovalbumin challenges. The anti-inflammatory effect of scutellarin has been well established [25, 58–60]. Here, we found that administration of scutellarin notably reduced the number of inflammatory cells in BALF and downregulated the concentrations of IgE, Th2 cytokines, eotaxin, and TGF-β1 in ovalbumin-challenged asthmatic mice, suggesting that scutellarin inhibited the ovalbumin-induced airway inflammation in mice.

In EMT, epithelial cells differentiate into myofibroblasts, providing the cells with migrative potential and initiating subepithelial fibrosis in airway remodeling. Dysfunctional EMT is responsible for mesenchymal cell generation, organ fibrosis, fibrotic tissue repair, and cancer metastasis [61–63]. Previous studies have shown that EMT is an important mechanism contributing to airway remodeling in severe refractory asthma [64, 65]. Increase in TGF-β1 levels can induce fibroblast activation into myofibroblasts by epithelial cells and promote the development of EMT [66]. In mice, ovalbumin can also induce EMT [67]. α-SMA is a myofibroblast specific marker. The myofibroblast cells can transform to smooth muscle cells with the extracellular matrix deposition, resulting in thickening of smooth muscle layer [68]. Here, we found that the expression of E-cadherin was decreased and the expression of N-cadherin and α-SMA was elevated in both cellular and animal models of asthma. The EMT inhibitory action of scutellarin has been previously documented [25, 69–71]. Scutellarin can also ameliorate pulmonary, myocardial, and cardiac fibrosis [25, 72–74]. In the current study, we found that scutellarin inhibited cell migration and also elevated E-cadherin expression and reduced N-cadherin and α-SMA expression in experimental asthma models, indicating that scutellarin can inhibit the EMT process by recovering E-cadherin expression and suppressing N-cadherin and α-SMA induction following asthma.

The process of EMT is associated with the TGF-β/Smad/MAPK pathways [75–78]. TGF-β1 stimulation can lead to the activation of Smad2/3 and MAPKs, resulting in tissue fibrosis and inflammation [79, 80]. Scutellarin can reduce the expression of TGF-β1, ERK, and p38 to alleviate interstitial fibrosis and cardiac dysfunction [35]. Scutellarin is also shown to inactivate the MAPK pathway to ameliorate osteoarthritis and cerebral ischemia/reperfusion injury [81–84]. Here, we found that scutellarin decreased the phosphorylation levels of Smad2/3, ERK, JNK, and p38 in TGF-β1-treated 16HBE cells and ovalbumin-challenged asthmatic mice.

Collectively, this study demonstrates that scutellarin can inactivate the Smad2/Smad3 and MAPK pathways to suppress the TGF-β1-induced fibrosis and EMT of 16HBE cells and relieve airway inflammation, remodeling, and EMT in ovalbumin-challenged asthmatic mice. We establish that scutellarin effectively improves the allergic asthma conditions by regulating the Smad2/Smad3 and MAPK pathways. To be honest, there are limitations to this study. First, the direct target of scutellarin has not been identified. Second, many other pathways, such as PI3K/Akt/mTOR pathway [85, 86], TLR4/MyD88/NF-κB pathway [87, 88], and Wnt/β-catenin pathway [89, 90], are involved in the regulation of airway inflammation and remodeling in asthma. More studies are required to the effect of scutellarin on these pathways. Third, the effect of scutellarin in ovalbumin-challenged asthmatic mice was examined at a single time point; additional time points need investigation. Despite these limitations, scutellarin treatment is a novel promising therapeutic strategy for allergic asthma. This study provides a theoretical basis for the application of scutellarin in the management of allergic asthma.

## Data Availability

The datasets used or analyzed during the current study are available from the corresponding author on reasonable request.
